# MRI and Additive Manufacturing of Nasal Alar Constructs for Patient-specific Reconstruction

**DOI:** 10.1038/s41598-017-10602-9

**Published:** 2017-08-30

**Authors:** Dafydd O. Visscher, Maureen van Eijnatten, Niels P. T. J. Liberton, Jan Wolff, Mark B. M. Hofman, Marco N. Helder, J. Peter W. Don Griot, Paul P. M. van Zuijlen

**Affiliations:** 1 Department of Plastic, Reconstructive and Hand Surgery, VU University Medical Centre, Amsterdam, 1081HV, the Netherlands, Amsterdam Movement Sciences, Amsterdam, The Netherlands; 2 Department of Oral and Maxillofacial Surgery/Oral Pathology – 3D InnovationLab, VU University Medical Center, 1081HV Amsterdam, The Netherlands; 3 Department of Physics and Medical Technology, VU University Medical Centre, Amsterdam, 1081 HV The Netherlands; 4 Department of Plastic, Reconstructive & Hand Surgery/Burn Centre, Red Cross Hospital, Beverwijk, 1942LE The Netherlands; 5 Association of Dutch Burn Centres, Beverwijk, 1942LE The Netherlands

## Abstract

Surgical reconstruction of cartilaginous defects remains a major challenge. In the current study, we aimed to identify an imaging strategy for the development of patient-specific constructs that aid in the reconstruction of nasal deformities. Magnetic Resonance Imaging (MRI) was performed on a human cadaver head to find the optimal MRI sequence for nasal cartilage. This sequence was subsequently used on a volunteer. Images of both were assessed by three independent researchers to determine measurement error and total segmentation time. Three dimensionally (3D) reconstructed alar cartilage was then additively manufactured. Validity was assessed by comparing manually segmented MR images to the gold standard (micro-CT). Manual segmentation allowed delineation of the nasal cartilages. Inter- and intra-observer agreement was acceptable in the cadaver (coefficient of variation 4.6–12.5%), but less in the volunteer (coefficient of variation 0.6–21.9%). Segmentation times did not differ between observers (cadaver P = 0.36; volunteer P = 0.6). The lateral crus of the alar cartilage was consistently identified by all observers, whereas part of the medial crus was consistently missed. This study suggests that MRI is a feasible imaging modality for the development of 3D alar constructs for patient-specific reconstruction.

## Introduction

The nose is a unique facial landmark that consists almost entirely of cartilage. Surgical reconstruction of cartilaginous defects caused by congenital disease (e.g. cleft palate^1^), trauma (e.g. burns^2^), or cancer^3^ remains a major challenge. The ultimate goal of surgical intervention in such cases is to restore the anatomical and physiological function of the nose (i.e. breathing).

The underlying nasal cartilages are of great importance for reconstruction^4, 5^ since they determine the shape and function of the nose. Reconstruction of the lower lateral cartilages can be performed using autologous septal cartilage^6, 7^, bone grafts^8^, or synthetic grafts^9^. However, these grafts often fail to mimic the three-dimensional (3D) morphology of native alar cartilage, are only available in certain sizes, and have to be carved by hand to fit the anatomical site^9^. Although recent advances in tissue engineering have shown interesting patient-specific methods for nasal cartilage reconstruction, accurate visualization of the complex 3D morphology remains challenging^10^.

To date, Magnetic Resonance Imaging (MRI) is the most commonly used imaging modality for the evaluation of cartilage *in situ*. MRI already plays an important role in the diagnosis of chondral lesions and determination of appropriate pharmacologic or surgical treatment and evaluation of such treatments^11^. MRI has also been used to differentiate soft tissues in the cleft region and their changes after surgery^12^. The major advantage of MRI is the adequate soft tissue contrast and its non-invasive nature (i.e. absence of radiation exposure). It may therefore be an adequate modality for imaging nasal cartilage to design and manufacture patient-specific cartilage constructs.

In this study, we aim to identify and provide a clinical step-by-step solution for patient-specific nasal alar reconstruction using MRI and additive manufacturing. First, the optimal MRI sequence for imaging nasal cartilage was determined in a cadaver study. Next, agreement and criterion validity of this MRI sequence was determined. Finally, the feasibility of using additive manufacturing to create morphologically accurate alar constructs for patient-specific reconstruction was assessed.

## Results

### Identification of alar cartilage and relevant MRI sequences

**Figure 1:**
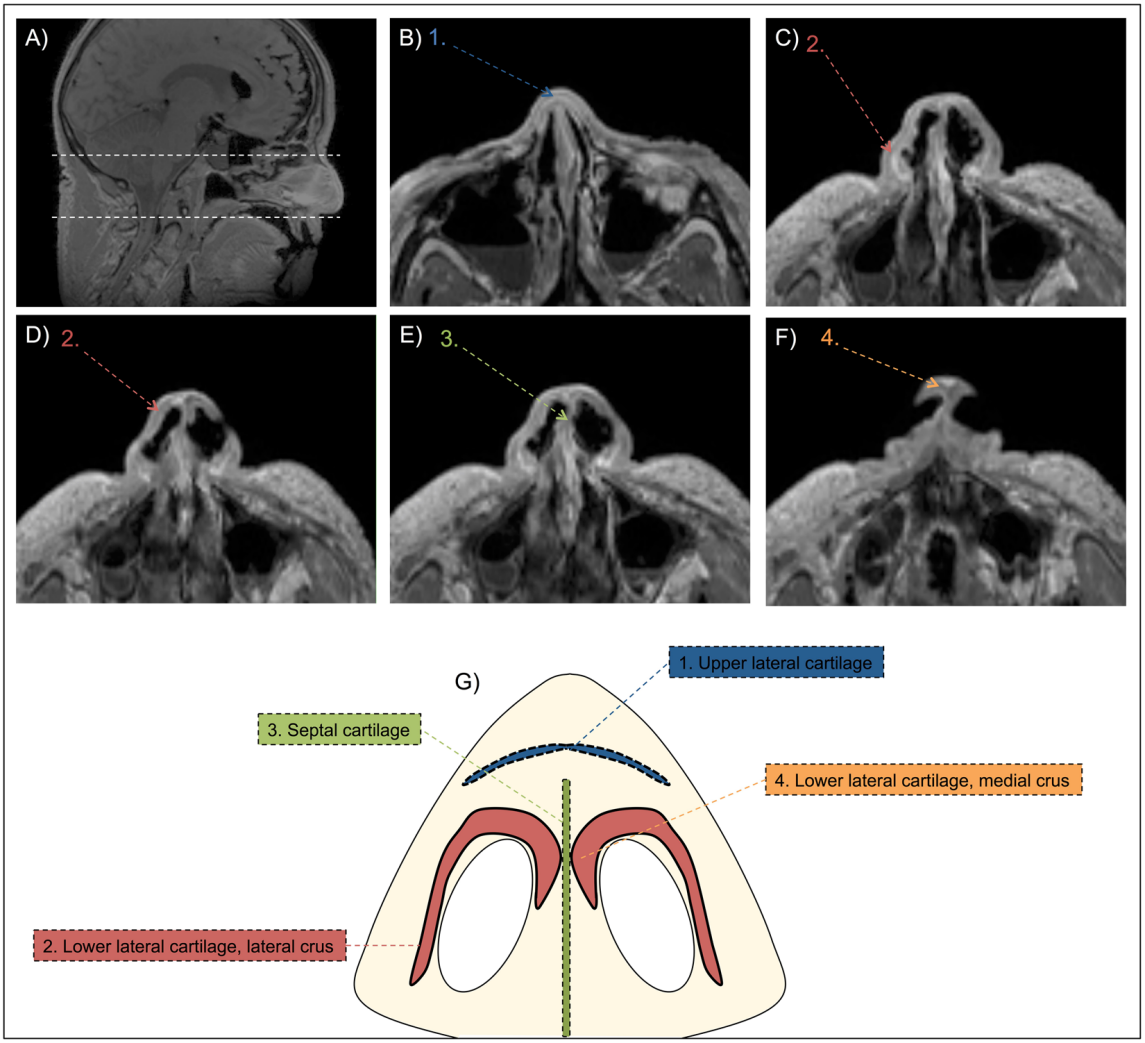
Depiction of nasal cartilages on 3T MRI (Spoiled GE) in Fix for Life Cadaver. (**A**) Sagittal scout MR scan. Area between white dotted lines indicates area of segmentation. (**B**) Axial reconstruction of the MR scan indicating upper lateral cartilage. (**C**) Axial reconstruction of the MR scan indicating lateral crus of the lower lateral cartilage. (**D**) Axial reconstruction of the MR scan indicating lower lateral cartilage. (**E**) Axial reconstruction of the MR scan indicating septal cartilage. (**F**) Axial reconstruction of the MR scan indicating medial crus of the lower lateral cartilage. (**G**) Illustration showing different cartilages in an axial slice of the nose. The numbers on the MRI correspond to the numbers on the illustration.

A range of different MRI sequences were available for cartilage imaging on the dedicated device (Table 1). After systematical assessment of all the sequences, the Spoiled GE sequence without fat saturation and a spatial resolution of 0.9 mm (see Table 1) provided the best contrast to noise ratio (i.e. best distinction between different layers) for the segmentation of the alar cartilage. The resulting MR images allowed depiction of the septal, upper lateral, and lower lateral cartilage on axial MRI slices as thin dark grey/black lines (Fig. 1). Starting superiorly from the nose (superior white dotted line, Fig. 1A), the first nasal cartilage to be identified was the upper lateral cartilage (Fig. 1B). Subsequently, by scrolling inferiorly, parts of the lateral crus of the lower lateral cartilage (Fig. 1C & D) and the septal cartilage (Fig. 1E) could be identified. The intersection of the right and left lower lateral cartilage (Fig. 1F) could be clearly identified more inferiorly (lower white dotted line, Fig. 1A) in the nose.

**Table 1:** Different MRI sequences tested for the identification of alar cartilage.

MRI Sequence	Echo Time [ms]	Repetition Time [ms]	Echo Train Length	Flip Angle [°]	Scan Time [min:sec]	Spatial Resolution x,y,z [mm]	2D/3D
CISS	3.1	7.9	1	40	02:28	0.4 × 0.4 × 1	3D
CISS	2.3	7.4	1	40	08:12	0.4 × 0.4 × 0.6	3D
FSE	34	1000	32	90	04:18	0.4 × 0.4 × 0.5	3D
FSE	14	2000	80	Variable	03:32	1.1 × 1.1 × 1	3D
FSE	19	2000	80	Variable	09:25	0.4 × 0.4 × 0.5	3D
MERGE	13	650	3	20	02:31	0.3 × 0.4 × 2.2	2D
Spoiled GE (FATSAT)	2.1	12	1	12	03:11	0.9 × 0.9 × 1	3D
Spoiled GE	2.3	7.1	1	12	04:46	0.5 × 0.5 × 0.5	3D
Spoiled GE	2.1	5.9	1	12	01:37	0.9 × 0.9 × 1	3D

CISS, Constructive Interference in Steady State; GE, Gradient Echo; FATSAT, Fat Saturation; MERGE, Multiple Echo Recombined Gradient Echo; FSE, Fast Spin Echo.

### Inter- and intra-observer agreement on MRI

There seemed to be no learning curve for manual segmentation of the alar cartilage (Suppl Table I). Average alar cartilage thickness (mm), surface area (mm^2^), and volume (mm^3^) in the cadaver were 2.12 ± 0.16 mm, 816 ± 52 mm^2^, and 570 ± 59 mm^3^, respectively (Table 2). In the volunteer these values were slightly higher at 2.29 ± 0.19 mm, 952 ± 80 mm^2^, and 630 ± 99 mm^3^, respectively (Table 2). In Table 2, the corresponding percent coefficient of variation (%CV) values are also shown in for each individual observer (intra-observer agreement) and all observers combined (inter-observer agreement). In both the cadaver and volunteer, the inter-observer agreement was lower for cartilage thickness (7.3% vs. 8.4% respectively) and surface area (6.4% vs. 8.4%) than for cartilage volume (10.3% vs. 15.7% respectively).

**Table 2:** Intra- and Inter-observer agreement of alar cartilage thickness (mm), surface area (mm^2^), and volume (mm^3^) on 3T MRI in a cadaver and volunteer by three independent observers.

Intra-observer agreement								Inter-observer agreement	
Measurement		Cadaver			Volunteer			Cadaver	Volunteer
		Observer 1	Observer 2	Observer 3	Observer 1	Observer 2	Observer 3	All Observers	
Thickness (mm)	Mean	2.15	2.13	2.09	2.45	2.31	2.13	2.12	2.29
	SD	0.1	0.2	2.1	0	0.1	0.2	0.2	0.2
	%CV	6.5%	11.3%	6.3%	0.6%	5.9%	10.4%	7.3%	8.4%
Surface area (mm^2^)	Mean	832	781	835	1022	861	972	816	952
	SD	38	60	57	27	50	47	52	80
	%CV	4.6%	7.6%	6.7%	2.6%	5.7%	4.8%	6.4%	8.4%
Volume (mm^3^)	Mean	590	550	569	720	570	599	570	630
	SD	64	69	62	64	125	27	59	99
	%CV	10.8%	12.5%	10.9%	8.9%	21.9%	4.5%	10.3%	15.7%

SD: Standard Deviation; %CV: percent coefficient of variation (SD/Mean). Measurements of thickness (mm), surface area (mm^2^), and volume (mm^3^) are shown for each observer (intra-observer agreement) and all observers combined (inter-observer agreement).

Intra- and inter-observer agreement differed between observers. For example, some observers were notably more consistent at segmenting cartilage thickness in the cadaver (6.5% and 6.3% CV vs. 11.3%), and cartilage volume in the volunteer (8.9% vs. 21.9% vs. 4.5%). In addition, one observer segmented cartilage thickness in the volunteer with low measurement error (%CV of 0.6%), while another observer with notably higher measurement error (%CV of 10.4%) (Table 2).

### Segmentation time for alar cartilage

Segmentation times of all observers for delineating the alar cartilage are shown in Table 2. On average, the time to segment, reconstruct, and export the cadaver STL files ranged from 7.3 ± 1.5 min to 10 ± 3 min. Similar segmentation times were found in the volunteer (Table 3). Segmentation times did not differ between observers in both cadaver (P = 0.36) and volunteer (P = 0.60).

**Table 3:** Average manual segmentation times (min) for one lateral alar cartilage in cadaver and volunteer by three individual observers.

Repeat	Cadaver			Volunteer		
	Observer 1	Observer 2	Observer 3	Observer 1	Observer 2	Observer 3
1	9 min	9 min	12 min	8 min	6.5 min	9 min
2	7 min	7.5 min	9.5 min	7 min	6.5 min	6 min
3	6 min	7 min	7 min	7 min	6 min	5.5 min
Mean time ± SD	7.3 ± 1.5	7.8 ± 1.0	9.5 ± 2.5	7.3 ± 0.6	6.3 ± 0.3	6.8 ± 1.9

### Validation of cartilage delineation on MRI

**Figure 2:**
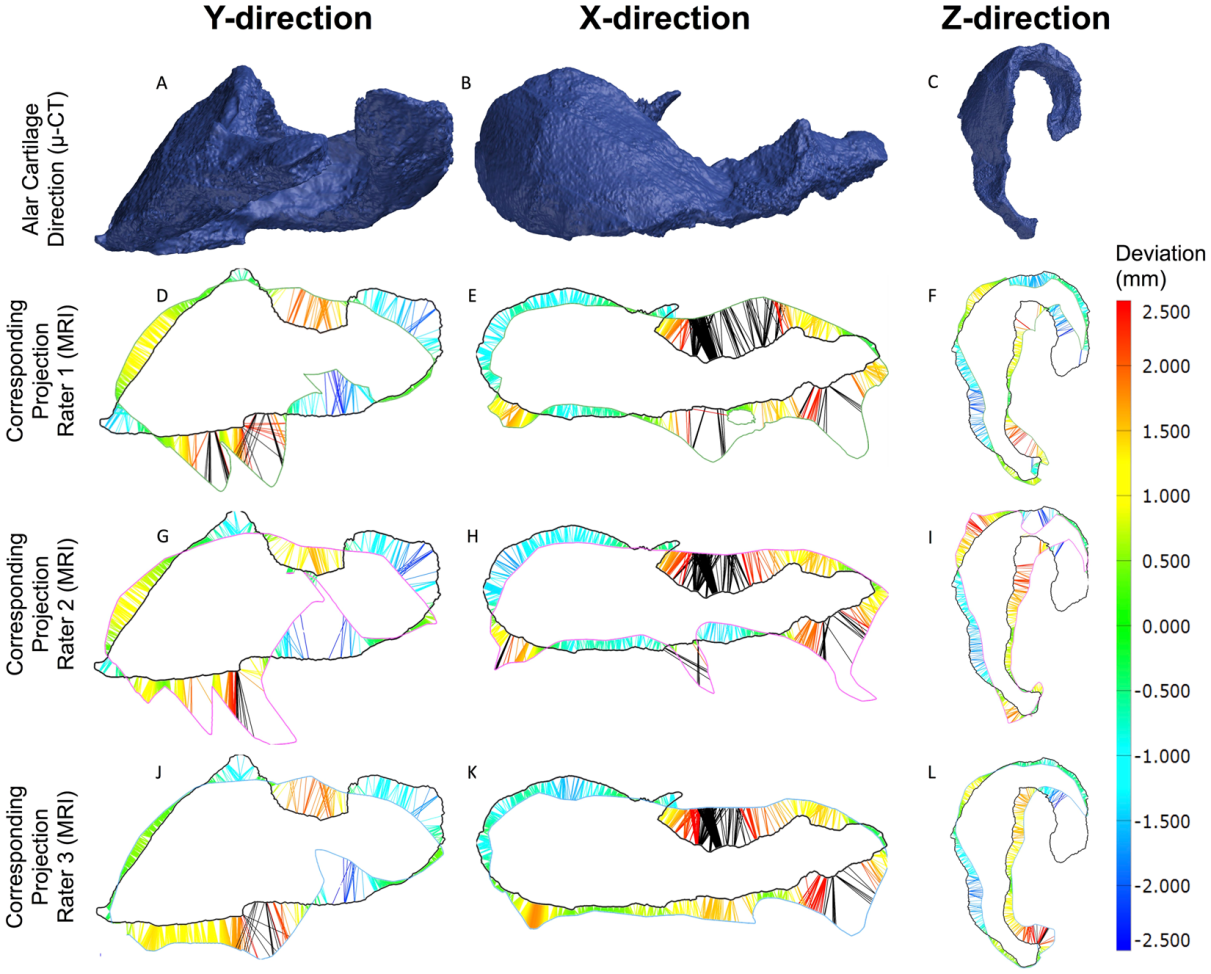
Criterion validity of the first alar cartilage segmentation on 3T MRI in a cadaver by three independent observers. (**A–C**) represent the alar cartilage STL model in three different directions as obtained from μ-CT. (**D–L**) shows the corresponding MRI projections and deviations from the μ-CT in millimeters (mm) for all three observers. (**D–F**) represent the projections for observer 1, (**G–I**) represent projections for observer 2, and (**J–L**) represent the projections for observer 3. The heat map shows the deviation in mm and its corresponding color on the projections. Black lines indicate a deviation of >2.5 mm.

Figure 2 shows the deviation (mm) of the first manual segmentation from the gold standard μ-CT (criterion validity) for all three observers. Overall, the maximum over- and underestimation was 2.5 mm (Fig. 2). The medial crus of the alar cartilage (see Fig. 1) was consistently missed on MRI by all three observers (Fig. 2F,I,L). The lateral crus (see Fig. 1) was consistently identified on the MRI by all three observers, with an overestimation of 2 mm in some parts (Fig. 2E,H,K). The absolute values for thickness (mm), surface area (mm^2^), and volume (mm^3^) for the native alar cartilage were 2.2 ± 0.3 mm, 938 mm^2^, 420 mm^3^ respectively. As shown in Table 2, the mean thickness (mm), surface area (mm^2^), and volume (mm^3^) for all observers were 2.12 ± 0.16 mm, 816 ± 52 mm^2^, and 570 ± 59 mm^3^.

### Additive manufacturing

**Figure 3:**
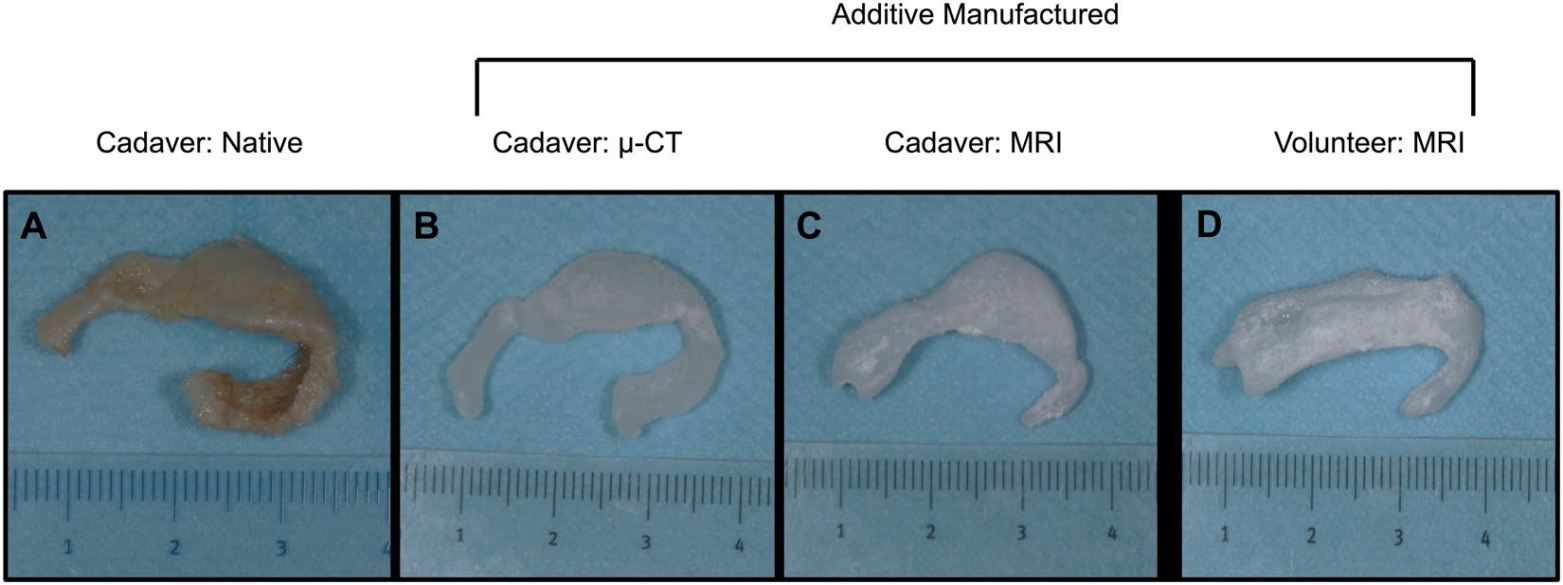
(**A**) Native alar cartilage obtained from cadaver, (**B**) additive manufactured silicon nasal alar construct based on μ-CT of native alar cartilage, (**C**) additive manufactured silicon alar construct based on MRI scan of cadaveric head, (**D**) additive manufactured silicon alar construct based on MRI scan of volunteer.

Additive manufactured alar constructs based on the μ-CT of the native cartilage showed a good anatomic resemblance to the native cartilage (Fig. 3B). The additive manufactured alar constructs derived using MRI also showed high fidelity to native cartilage for the lateral crus, but less so for the medial crus (Fig. 3C, one manufactured construct shown). The manufactured alar construct from the volunteer showed a lateral and medial crus. The medial crus in the volunteer was less well defined than in the cadaveric construct (Fig. 3D), but this can be explained by anatomical variance.

## Discussion

Reconstruction of complex nasal deformities remains a major challenge. Knowledge of the patient-specific nasal anatomy can aid the fabrication of tailored nasal constructs using additive manufacturing and tissue engineering. The results of this study demonstrate that MRI is a valid method to visualize alar cartilage and can therefore be used to additive manufacture patient-specific alar constructs.

The %CV values observed for alar cartilage thickness (mm), surface area (mm^2^), and volume (mm^3^) in the cadaver and volunteer were mostly equal or less than 10%, meaning that there is a general inter- and intra-observer agreement with low measurement error in repeated measurements (Table 2). Clinically speaking, this indicates that one observer can repeatedly identify the same alar cartilage on the MRI using manual segmentation. However, %CV values for volume (mm^3^) were generally higher than for cartilage thickness (mm) and cartilage surface area (mm^2^) (Table 2), indicating that the correct volume of the cartilage was harder to identify on the MRI. In this context, volumetric variations can be explained by overestimation of the cartilage on the MRI (Fig. 2), which would not have any clinical implications as excess material can be removed by the surgeon.

Compared to the cadaver, manual segmentation of the alar cartilage in the volunteer proved a lot more difficult judging from the variation in cartilage thickness, surface area, and volume (Table 2). The most likely explanation is that involuntary movement of the nostrils during MRI may have caused blurring of the images in the volunteer. Motion is the most common cause of artefacts in MR imaging and can be minimized by educating the patient or intravenous sedation^13^. This is in agreement with a previous study by Kleinheinz & Joos^12^ who acquired good MR images of the nasal cartilage in children by anesthetizing the children prior to imaging.

Manual segmentation of the alar cartilage could be performed quickly, with an average of time approximately seven minutes in both the cadaver and volunteer. There was no significant difference in segmentation time between the observers (Table 3) and there seemed to be no relationship between average segmentation time and measurement error (Tables 2 and 3). Ultimately, this means that manual alar cartilage segmentation can be repeated predictably in clinical settings.

In order to evaluate the criterion validity of MRI-derived STL models of the alar cartilage all cadaveric STL models were geometrically compared to the μ-CT-derived STL model of the dissected cadaver cartilage (gold standard). A similar method has previously been used to test the precision and accuracy of MRI of ear cartilage^14^. Dissection of the cadaveric alar cartilage demonstrated a complex morphology (Fig. 3). The lateral crus of the cadaveric alar cartilage was overestimated by all observers, while the medial crus was underestimated by approximately 2 mm by all observers (Fig. 2F,I,L). As mentioned previously, the surgeon can correct for overestimation. However, underestimation of the medial crus cannot be corrected for. Although this might seem a problem for the production of patient-specific alar constructs, the medial segment of the lateral crus, which was identified correctly on MRI, is considered the primary component of the nasal lobule that defines its shape, size, and position^15^. Therefore, identification of the whole medial crus might not be as clinically relevant.

**Figure 4:**
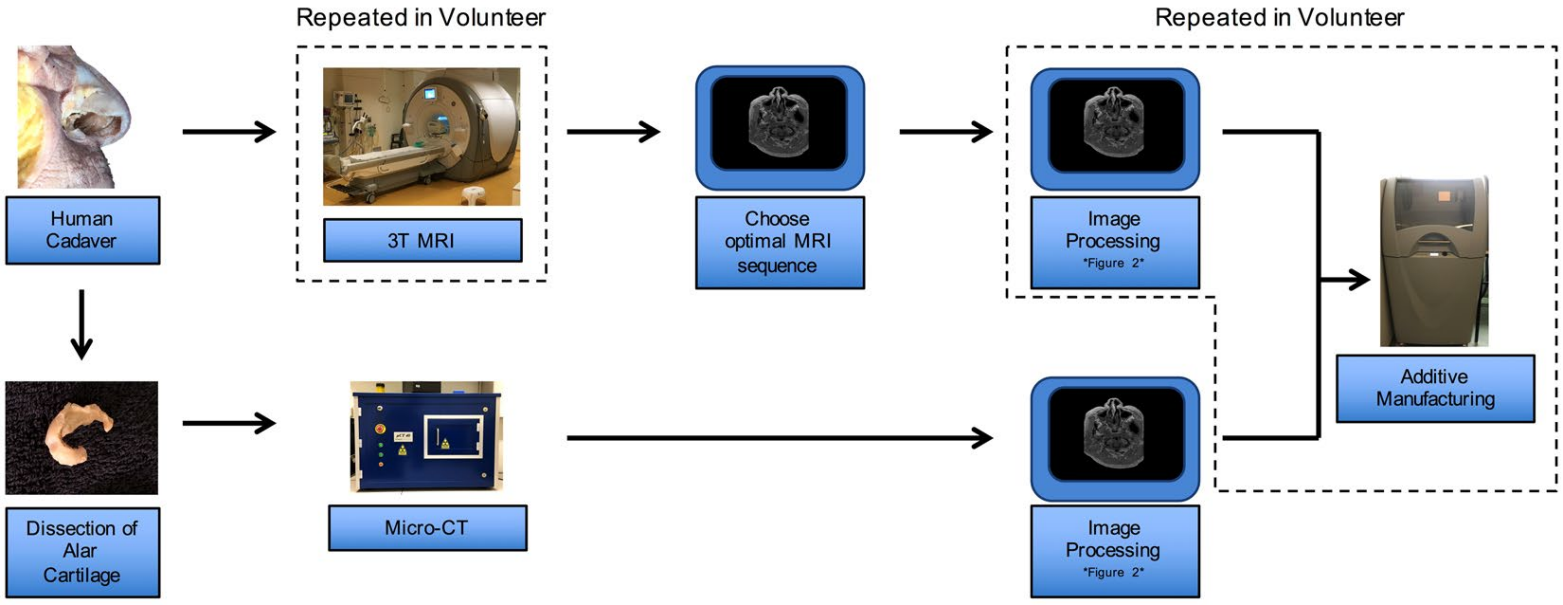
Schematic of the steps undertaken in this study. A Fix for Life cadaver head was used to identify alar cartilage on MRI. 3T MRI was used to identify the MRI sequence that provided the best visualization for alar cartilage segmentation. The right alar cartilage was dissected from the cadaveric head and scanned using micro-CT. The resulting images were then processed in imaging software (see Fig. 5) for additive manufacturing. 3T MRI, Image processing, and additive manufacturing were repeated in a volunteer (squared boxes). 3T, 3 Tesla; micro-CT, micro-Computed Tomography.

**Figure 5:**
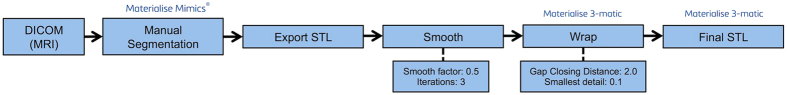
Image processing steps and software packages required to obtain the final STL from DICOM data. DICOM data is obtained from the MRI, manually segmented in Materialise Mimics, and exported as STL to Materialise 3-matic. Here the construct is smoothed and wrapped and again exported as STL file. DICOM, Digital Images and Communications in Medicine; STL, Standard Tessellation Language file.

In light of the abovementioned findings, it is important to realize that the clinical application of techniques such as MRI imaging (spatial resolution of 0.9 mm) and additive manufacturing (see Figs 4 & 5) introduce minor cumulative errors in the thickness (mm), surface area (mm^2^), and volume (mm^3^) of the alar cartilage. These results are in accordance with previous findings, where MRI-derived STL models and additive manufacturing introduced errors of 1.5 mm and 0.5 mm respectively^16^. Therefore, in a clinical setting, an error margin of <2 mm can be considered satisfactory.

The present study demonstrates that MR images of the nose can be used to additively manufacture patient-specific silicon alar constructs (Fig. 3). The proposed method can be applied to a wide range of patients and can also function as a basis for tissue engineering of alar cartilage^10^. Clinical MR imaging in combination with additive manufacturing could ultimately lead to patient-specific alar constructs that can be used for facial reconstruction (e.g., burn reconstruction or orofacial cleft repair). To the best of our knowledge, this is the first study that has used MRI to manufacture patient-specific alar constructs.

## Conclusion

The current study demonstrated that manual segmentation of alar cartilage on 3 Tesla MRI is a repeatable and valid method to produce alar constructs for patient-specific reconstruction. This method may be used to produce synthetic 3D-printed patient-specific alar implants, or may provide a strategy to produce alar scaffolds for tissue engineering. However, it should be noted that the quality of the overlying skin is just as important for reconstruction as the underlying implant.

## Methods

*Additional data that is not included in this manuscript or in the supplementary material can be obtained from the corresponding author upon reasonable request*.

A female human cadaver head (age 76) embedded in “Fix for Life” embalming liquid^17^ was obtained from the department of Anatomy at the VU University Medical Centre (VUmc, Amsterdam, the Netherlands) in full accordance with Article 1 of the Dutch law on funeral services^18^ and European legislation. In addition, ethical approval for use of the cadaveric head was acquired through the Medical Ethical Committee of the VU University Medical Centre (Ref. 2016401). The head was imaged on a 3 Tesla whole body MRI scanner (Discovery™ MR750; GE Healthcare, Chicago, IL, USA) equipped with a thirty-two-channel head radiofrequency receiver coil. A series of MRI sequences (Table 1) was tested in order to determine which sequence provided the best visualization of the alar cartilage. In a second step, this sequence was tested on a healthy volunteer (Fig. 4) under supervision of a fully trained clinical physicist, and in accordance with all relevant guidelines and regulations. The experimental protocol was approved by the VU University Medical Centre. The volunteer was asked to provide written informed consent prior to study participation and for publication of the resulting images.

Following imaging using different MRI sequences, all cadaver MRI scans were saved as Digital Imaging and Communications in Medicine (DICOM) files and imported into Mimics Medical software (version 19.0; Materialise, Leuven, Belgium) (Fig. 5). The acquired DICOM files were then systematically assessed on the computer for the optimal alar cartilage visualization by one observer. One DICOM file with the corresponding MRI sequence was subsequently selected for re-imaging in the cadaver. This sequence was also used on the female volunteer.

### Image processing: DICOM to STL conversion

The resulting two MRI scans (cadaver & volunteer) were subsequently manually segmented by three independent observers who delineated the alar cartilage on the axial slices of the MRI. The segmentation procedure was repeated after a ten-day interval for a total of three times (n = 3). The observers were blinded for their own results and the results of each other. All segmented structures were converted into 3D surface models, hence standard tessellation language (STL) models, and exported to 3-matic software (version 11.0; Materialise, Leuven, Belgium). In 3-matic, all STL models were subjected to smoothing and wrapping (Fig. 5). Thickness (mm), surface area (mm^2^), and volumetric (mm^3^) measurements of all STL models were performed using GOM inspect® software (GOM inspect v8, GOM mbH, Braunschweig, Germany).

### Dissection and Micro-CT

Following MRI of the cadaver head, the right alar cartilage was carefully dissected with a surgical knife and forceps by an experienced plastic surgeon. The dissected cartilage was then fixed in synthetic foam and placed vertically in a polyetherimide holder. The alar cartilage was scanned at 72.0 μm isotropic voxel size, 70 kV source voltage, and 114 μA current using a high resolution micro-CT system (μCT 40, Scanco Medical AG, Basserdorf, Switzerland). The DICOM images obtained from the micro-CT were reconstructed in Mimics software and exported as an STL file. The cadaver STL file was used as the gold standard in this study.

### Criterion Validity

Criterion validity is the degree to which the performance of, in this case manual segmentation, is an adequate reflection of the ‘gold standard’^19^. Criterion validity was determined by geometrically comparing the cadaveric MRI-derived STL models with the gold standard STL model (μ-CT) using silhouettes in three different directions in GOM Inspect® software. Deviation from the gold standard μ-CT (mm) was calculated using coloured whiskers.

### Additive manufacturing

Nasal alar molds were designed from the alar cartilage STL files obtained from the μ-CT and MRI scans (cadaver & volunteer) using 3-matic software. The molds of the average segmentations (n = 3) for each observer (n = 3) were printed in triplicate on a Zprinter 250 inkjet powder printer (3D Systems Inc., Rock Hill, SC) giving a total number of 21 molds, including three molds based on the STL file obtained from the μ-CT. Following printing, the alar molds were impregnated with salt and oven-dried for three hours prior to injection with Dragon Skin® silicone 30. The construct was air-dried for another 12 hours and powder was washed out using running tap water leaving a flexible silicon alar construct.

### Statistical analysis

Statistical analysis was performed using SPSS software (SPSS® Statistics 22.0 for Windows; SPSS Inc., Chicago, IL, USA). Agreement parameters assess how close the results of the repeated measure are, by estimating the measurement error in repeated measures^20^. In order to determine the intra- and inter-observer agreement in both the cadaver and volunteer, the mean, SD, and percent coefficient of variation (%CV) were calculated for alar thickness (mm), volume (mm^3^), and surface area (mm^2^). One-way analysis of variance (ANOVA) was used to determine differences in segmentation times between the three observers. Criterion validity, as mentioned above, was only calculated for the cadaveric MRI-derived STL model. Values of *p* < *0.05* were considered statistically significant.

## Electronic supplementary material

Supplementary Table I
