# Supplementary material for: MRI and Additive Manufacturing of Nasal Alar Constructs for Patient-specific Reconstruction
Source: Sci Rep. 2017 Aug 30;7:10021. doi: 10.1038/s41598-017-10602-9 (PMC5577227; doi:10.1038/s41598-017-10602-9)
Supplement: Supplementary file 1 — Supplementary Table I [file 41598_2017_10602_MOESM1_ESM.pdf]

MRI and Additive Manufacturing of Nasal Alar Constructs for Patient-specific Reconstruction

Dafydd O. Visscher, Maureen van Eijnatten, Niels P.T.J. Liberton, Jan Wolff, Mark B.M. Hofman, Marco N. Helder, J. Peter Don Griot, Paul P.M van Zuijlen

Supplementary Table I. Raw data on cartilage thickness (mm), surface area (mm^2), and volume (mm^3) on 3.0-T MRI in a cadaver and volunteer by 3 observers.

Cartilage Thickness (mm)	*Cadaver (MRI)*			*Gold standard (μ-CT)*	*Volunteer (MRI)*		
**Observer 1**	**Repeat 1**	**Repeat 2**	**Repeat 3**		**Repeat 1**	**Repeat 2**	**Repeat 3**
**Measure 1**	1.9	1.5	2.1	1.9	2.1	2.2	2.2
**Measure 2**	2.2	2.6	2.5	2.5	2.2	2.2	2.1
**Measure 3**	2.0	2.2	2.4	2.1	3.0	3.0	3.1
**Mean**	2.0	2.1	2.3	2.2	2.4	2.5	2.5
**SD**	0.2	0.6	0.2	0.3	0.5	0.5	0.5
**Observer 2**							
**Measure 1**	2.0	1.5	2.0	1.9	1.8	2.3	2.4
**Measure 2**	1.8	2.2	2.8	2.5	2.3	2.6	1.7
**Measure 3**	2.1	2.3	2.4	2.1	2.7	2.4	2.5
**Mean**	2.0	2.0	2.4	2.2	2.3	2.5	2.2
**SD**	0.2	0.4	0.4	0.3	0.4	0.2	0.4
**Observer 3**							
**Measure 1**	2.0	1.8	1.9	1.9	1.8	2.0	2.3
**Measure 2**	1.8	2.1	2.1	2.5	1.9	2.2	1.3
**Measure 3**	2.1	2.4	2.6	2.1	2.4	3.0	2.3
**Mean**	2.0	2.1	2.2	2.2	2.0	2.4	2.0
**SD**	0.1	0.3	0.4	0.3	0.3	0.5	0.6
**Cartilage Surface Area (mm^2)**							
**Observer 1**	791	841	864	938	998	1016	1050
**Observer 2**	739	754	849	938	807	871	905
**Observer 3**	772	882	849	938	1019	972	925
**Cartilage Volume (mm^3)**							
**Observer 1**	551	555	664	420	700	669	792
**Observer 2**	482	547	620	420	427	626	657
**Observer 3**	500	621	586	420	575	628	594

Raw data, mean, and standard deviation (SD) are shown for manual segmentations by all 3 observers. The gold standard (μ-CT) is also shown in the middle column. Measures 1-3, as shown in the first column for cartilage thickness (mm), indicate measurements taken at 3 points on the alar cartilage. μ-CT, micro-computed tomography; MRI, magnetic resonance imaging; SD, standard deviation
